# Special Issue “DNA Replication/Repair, and the DNA Damage Response in Human Disease”

**DOI:** 10.3390/genes14040893

**Published:** 2023-04-11

**Authors:** Dong Zhang, Kristin A. Eckert, Marietta Y. W. T. Lee

**Affiliations:** 1 Department of Biomedical Sciences, College of Osteopathic Medicine, New York Institute of Technology, Old Westbury, NY 11568, USA; 2 Center for Cancer Research, New York Institute of Technology, Old Westbury, NY 11568, USA; 3 Gittlen Cancer Research Laboratories, Department of Pathology, Penn State University College of Medicine, Hershey, PA 17036, USA; 4 Department Biochemistry and Molecular Biology, New York Medical College, Valhalla, NY 10595, USA

Mutations of numerous genes involved in DNA replication, DNA repair, and DNA damage response (DDR) pathways lead to a variety of human diseases, including aging and cancer [1]. With the approval of PARP inhibitors in treating certain cancers carrying mutations in the *BRCA1* and/or *BRCA2* genes [2], targeting the DNA metabolism has become an important strategy in drug discovery. This Special Issue of *Genes* highlights the critical components of DNA replication, DNA repair, and DDR, as well as their potential roles in human diseases.

The first fraction of papers in this Special Issue focuses on the canonical and non-canonical roles of DNA polymerases and their associated proteins. In a research article, Njeri and her colleagues report the identification of lysine acetylation of multiple subunits of human DNA polymerase delta (δ) [3], which is essential to the synthesis of the lagging strands during the S phase [4,5]. Using a variety of in vitro and in vivo assays, they demonstrate that lysine acetylation is crucial for the functional properties of DNA polymerase δ. Most importantly, acetylation stimulates the ability of the polymerase to resolve secondary structures that are present on the template strand. Another DNA polymerase, DNA polymerase eta (η), belongs to a family of DNA polymerases, of which the major function is to bypass various genomic lesions, such as the thymidine dimers induced by ultraviolet (UV). Therefore, these are called translesion synthesis (TLS) DNA polymerases. In a review article, Eckert discusses the non-canonical functions of DNA polymerase η, with an emphasis on its role in facilitating DNA synthesis through the difficult-to-replicate genomic regions, including telomeres, common fragile sites (CFS), centromeres, rDNA loci [2,6], and the potential roles for its reverse-transcriptase activity [7]. In addition to its canonical function in synthesizing the lagging strands, DNA polymerase δ is also an essential component of a unique homology-dependent repair (HDR) pathway, called break-induced replication (BIR) [8,9]. To systematically identify the suppressors of BIR, Riders and colleagues performed a genome-wide shRNA screening using an intricately designed reporter [10]. In addition to identifying many known DNA replication, DNA repair, and DDR genes, they also identified a few unexpected suppressors, such as COPS2, one of the key subunits of COP9 signalosome. Further characterization of these new suppressors of BIR will certainly shed new lights on the molecular mechanism of BIR. Exactly twenty years ago, POLDIP3 was identified as one of the DNA-polymerase-δ-interacting proteins [11]. Subsequently, the Lee lab demonstrated that POLDIP3 has a critical role in regulating the enzymatic activity of DNA polymerase δ [4]. In a review article, Singh and colleagues discuss the biochemical and biological functions of POLDIP3, not only in the context of DNA metabolism, but also its role in various RNA metabolic pathways [12]. In partnering with DNA polymerase δ, the flap endonuclease 1 (FEN1) has a significant function in Okazaki fragment maturation [5] and in processing DNA–RNA hybrids, i.e., R-loops [13]. In a research article, Laverde and colleagues use a variety of intricate biochemical assays establishing that FEN1 cleaves the RNA flap in coordination with APE1 to facilitate the resolution of R-loops through DNA base excision repair, providing mechanistic insights into the mechanism behind this novel function of FEN1 [14].

The second fraction of papers in this Special Issue focuses on the role of DNA replication, DNA repair, and DDR in maintaining the integrity of telomeres, the dysregulation of which has a great impact on a variety of human diseases, including aging and cancer [15]. Human telomeres are unique genomic sequences found at the end of each chromosome [16]. Maintaining telomeres is vital for the health of a mammalian cell, as excessively shortened telomeres induce cell cycle arrest, senescence, and cell death. In actively proliferating cells, including cancers, two telomere maintenance mechanisms exist: (1) continued activation of telomerase; and (2) the alternative lengthening of telomeres (ALT). Mutations in many components of the human telomerase complex, including hTERT, lead to telomere biology disorders (TBDs) or telomeropathies [17]. In a research article, Welfer and colleagues characterized TBD-related TERT variants using single-turnover kinetics and computer simulation [18]. Their findings shed new light on how these variants potentially affect the catalytic functions of telomerase. Telomeres are genomic regions prone to replication stress, and one strategy to minimize the deleterious effects of replication stress-induced DNA damage is to activate a unique DNA repair process in mitosis, called DNA synthesis in mitosis (MiDAS). In a review article, Barnes and colleagues discussed the molecular mechanism of MiDAS in the context of telomeres [19]. Additionally, since both POLDIP3 and BIR have been implicated in the ALT pathway, the two articles written by Singh et al. and Riders et al. are valuable resources to further the understanding of the ALT pathway [10,12]. Finally, Batista and colleagues summarized the biology and therapeutics of Hutchinson-Gilford Progeria Syndrome (HGPS), one of the premature aging syndromes [20]. HGPS is mainly caused by a mutation in the *LMNA* gene, which encodes both lamin A and lamin C, two major components of nuclear lamina. Interestingly, cells derived from HGPS patients manifest a variety of defects in DNA repair, DDR, and telomere biology, which may account for their various clinical manifestations.

Chromatin reassembly and remodeling are intimately connected to DNA replication, DNA repair, and DDR. In a review article, Belousova and Lavrik summarize the potential role of PARP1, one of the 17 enzymes catalyzing the ADP ribosylation reaction [21] in ATP-independent nucleosome reorganization during DNA repair and DDR [22]. Finally, high glucose levels have been shown to induce DNA damage and genome instability [23]. In a research article, Rahmoon and colleagues investigate the connection between high glucose levels and DDR in normal mammary epithelial cells and triple-negative breast cancer cells [24]. Their research indicates new findings relating to the mechanism of high glucose in affecting DNA repair and DDR.

## Data Availability

Not applicable.
